# A Review of Wireless Charging Solutions for FANETs in IoT-Enabled Smart Environments

**DOI:** 10.3390/s26030912

**Published:** 2026-01-30

**Authors:** Nelofar Aslam, Hongyu Wang, Hamada Esmaiel, Naveed Ur Rehman Junejo, Adel Agamy

**Affiliations:** 1 School of Information and Communication Engineering, Dalian University of Technology, Dalian 116024, China; whyu@dlut.edu.cn; 2 Electrical Engineering Department, College of Engineering, King Khalid University, Abha 61411, Saudi Arabia; hesmaiel@kku.edu.sa; 3 Department of Computer Engineering, The University of Lahore, Lahore 54000, Pakistan; naveed.rehman@dce.uol.edu.pk; 4 Faculty of Engineering, Department of Electrical Engineering, Aswan University, Aswan 81542, Egypt; a.f.agamy@aswu.edu.eg

**Keywords:** wireless power transfer, unmanned aerial vehicle, flying ad hoc network, internet of things, UAV trajectory optimization

## Abstract

Unmanned Aerial Vehicles (UAVs) are emerging as a fundamental part of Flying Ad Hoc Networks (FANETs). However, owing to the limited energy capacity of UAV batteries, wireless power transfer (WPT) technologies have recently gained interest from researchers, offering recharging possibilities for FANETs. Based on this background, this study highlights the need for wireless charging to enhance the operational endurance of FANETs in Internet-of-Things (IoT) environments. This review investigates WPT power replenishment to explore the dynamic usage of UAVs in two ways. The former is for using a UAV as a mobile charger to recharge the ground nodes, whereas the latter is for WPT applications in in-flight (UAV-to-UAV) charging. For the two research domains, we describe the different methods of WPT and its latest advancements through the academic and industrial research literature. We categorized the results based on the power transfer range, efficiency, wireless charger topology (ground or in-flight), coordination among multiple UAVs, and trajectory optimization formulation. A crucial finding is that in-flight UAV charging can extend the endurance by three times compared to using standalone batteries. Furthermore, the integration of IoT for the deployment of a clan of UAVs as a FANET is rigorously emphasized. Our data findings also indicate the present and future forecasting graphs of UAVs and IoT-integrating UAVs in the global market. Existing systems have scalability issues beyond 20 UAVs; therefore, future research requires edge computing for WPT scheduling and blockchains for energy trading.

## 1. Introduction

**Figure 1:**
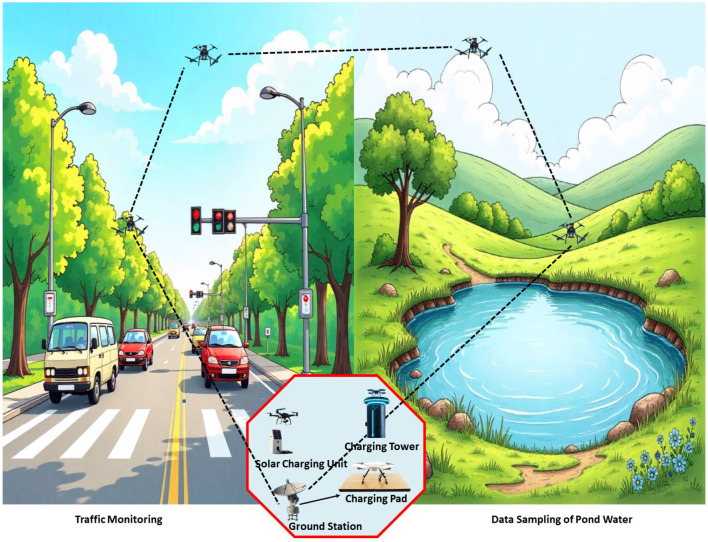
Wireless recharging in a Flying Ad Hoc Network with an IoT smart environment.

Flying Ad Hoc Networks (FANETs) are self-organizing networks formed by Unmanned Aerial Vehicles (UAVs) that communicate wirelessly in a dynamic topology infrastructure [1,2]. FANETs are rapidly gaining prominence across various sectors, including smart cities [3,4,5], agriculture [6], logistics [7], healthcare [8,9], military surveillance [10], and disaster response [11,12,13]. As mobile nodes are capable of flexible deployment and autonomous operation in adverse and dynamic conditions, FANETs play a crucial role in modern IoT ecosystems by enabling real-time data collection, infrastructure monitoring, and situational awareness [14,15]. Unlike Mobile Ad Hoc Networks (MANETs) or Vehicular Ad Hoc Networks (VANETs), FANETs operate in 3D airspace, experience more frequent topology changes, and require fast response times [16], as in a FANET, these interconnected small UAVs are capable of communicating data between each other and with the base station (BS) [17]. An IoT-integrated FANET application is shown in Figure 1.

However, their operational efficiency is significantly dependent on their limited onboard battery capacity, making energy management a central challenge in the design and deployment of FANETs [18]. Ineffective energy management in traditional FANETs results in limited onboard energy, leading to the mismanagement of mobility, latency constraints, and bandwidth limitations, all of which significantly affect vehicle capabilities. Most traditional UAVs operate on a confined battery life (typically 20–60 min) [19], restricting the mission duration and radius from the base within which the vehicle can operate. Traditional battery-dependent models lead to frequent interruptions, reduced mission times, scalability issues, and logistical challenges associated with recharging or replacing batteries [20]. Wireless charging technologies, ranging from static ground-based systems to dynamic in-flight energy transfer, are emerging as promising solutions for extending the operational endurance of UAVs in FANETs when managed intelligently [21].

Wireless power transfer (WPT) technologies have become increasingly relevant in overcoming the energy limitations faced by UAVs, particularly in the context of FANETs operating within smart environments. Several WPT methods have been explored for UAV applications, each with distinct operational characteristics [22]. Inductive coupling offers high efficiency over short distances and is well-suited for static charging stations, such as landing pads, although it requires precise alignment [23]. Resonant inductive coupling builds on this by allowing more flexible alignment and greater transmission distances, making it more adaptable to real-world UAV deployment scenarios [22,23]. Microwave power transfer (MPT) enables in-flight charging through directed electromagnetic beams, offering the potential for continuous operation, although it requires accurate beam tracking and raises safety concerns [24]. Similarly, laser-based power transfer enables high-energy wireless charging over long distances using focused laser beams and onboard photovoltaic receivers, supporting uninterrupted flight but requiring a clear line of sight and presenting challenges related to safety and weather sensitivity [25].

By enabling in-flight energy replenishment, they reduce the need for human intervention and support longer or continuous UAV operations [26]. Nevertheless, without intelligent coordination, these systems may lead to incompetence in charge scheduling, increased UAV downtime, and unbalanced energy consumption across the networks. IoT integration is a critical enabler, particularly in dynamic environments with adverse conditions that can significantly impact system performance [27]. When combined with IoT-enabled communication, sensing, and control frameworks, WPT technologies can be optimally managed to ensure balanced energy distribution, predictive maintenance, and mission-aware scheduling. Ultimately, this enhances the endurance, resilience, and autonomy of FANET operations [28].

Coordinated energy management plays a critical role in maintaining operational continuity and system resilience in swarm and multi-UAV systems within FANETs. One key strategy is coordinated wireless charging, in which UAVs collaboratively manage energy resources through structured scheduling and role-based behaviors [29]. For instance, master–slave refueling models allow certain UAVs with higher energy reserves or designated energy-harvesting capabilities to temporarily act as mobile charging nodes for others [5], reducing the need for all units to return to static charging stations. Additionally, IoT-enabled intellectual scheduling algorithms and real-time data sharing can optimize flight paths, assign charging priorities, and coordinate mission handovers to minimize downtime and energy waste. Such coordination is particularly important in time-sensitive or large-scale missions in which continuous aerial coverage is essential. By leveraging swarm intelligence and distributed decision-making, coordinated charging not only enhances the endurance of individual UAVs but also improves the overall efficiency, robustness, and autonomy of FANETs [4]. IoT technologies, including sensor networks, cloud-based analytics, and low-latency communication protocols, can facilitate the smart coordination of wireless charging processes [30]. Through real-time data acquisition, energy-aware task allocation, and predictive maintenance, IoT platforms enable adaptive energy management that aligns with optimizing charging schedules, dynamic mission coordination requirements, and environmental conditions [31,32] with system design.

Most existing studies either focus on wireless charging techniques or IoT-based UAV coordination without holistically addressing how IoT can enhance energy management and smart charging in FANETs [33]. This lack of incorporation in the literature highlights the need for a systematic review to assess the current state of the art, synthesize existing approaches, and identify opportunities for future innovations.

This review explores how the convergence of IoT and wireless charging enhances the energy efficiency, autonomy, and scalability of FANETs. It focuses on the current trends of on-ground and in-flight wireless recharging of UAVs, as well as the deployment issues of wireless recharging in FANETs. In addition, numerous challenges associated with deploying WPT within FANETs have been explored, including energy efficiency, range alignment, power delivery, infrastructure scalability cost, security, and privacy risks. The scope of this study comprises a discussion of wireless charging mechanisms, IoT-based control frameworks, UAV trajectory optimization, and future research directions at the intersection of FANETs, IoT, and wireless power transfer.

The main objective of this systematic literature review is to investigate how IoT technologies support and enhance wireless charging for FANETs in various applications. To guide this review, the following research questions (RQs) were addressed based on its objectives:

- What are the existing methods and architectures for wireless charging technologies for IoT-enabled FANETs? How do they perform in testing compared with the traditional charging of FANETs?
- How has the WPT evolved to recharge UAVs and FANETs as well?
- What are the existing systems and demonstrations of WPT in UAVs?
- What is the role of IoT in optimizing UAV trajectories that are employed as wireless chargers in FANETs?
- What are the current challenges, limitations, and open research gaps in deploying wireless charging in FANETs?
- How is IoT integrated into FANETs for energy management and coordination?

The key contributions of this review are as follows:

- This review provides a comprehensive synthesis of existing research on the integration of IoT technologies with wireless charging solutions for FANETs. By bridging diverse but interconnected fields, including wireless networking, energy management, UAV control, and smart environments, a multidisciplinary perspective on this state-of-the-art technology can be provided.
- It also identifies and classifies the primary technological approaches and system architectures currently employed in the field. In doing so, it highlights application-specific innovations across a range of domains, including precision agriculture, disaster response, and urban infrastructure. These findings demonstrate the practical relevance and growing interest in deploying IoT-enabled FANETs for energy-intensive missions in real-world scenarios.
- Furthermore, this review critically examines the current limitations of the literature, drawing attention to persistent research gaps related to energy efficiency, interoperability, scalability, and cybersecurity. By analyzing these challenges, we provide a foundation for future research to address the pressing technical and operational barriers.
- In addition to mapping the existing research landscape, this review offers comparative evaluations and design insights that can inform the development of more integrated and efficient systems in the future. The study concludes with strategic recommendations for advancing the integration of IoT and wireless charging in dynamic FANET environments. This study serves as a valuable reference for researchers and practitioners seeking to develop resilient, scalable, and autonomous UAV systems capable of sustained operation in energy-constrained and mission-critical settings.

The remainder of this paper is organized as follows. Section 2 outlines the methodology of the systematic literature review. Section 3 elaborates on the fundamental features of wireless recharging. Section 4 focuses on the research challenges of WPT for FANETs. Section 5 discusses the deployment issues of wireless recharging in FANETs. Section 6 presents graphical representations of the global UAV market and the implementation of wireless recharging in UAVs. Section 7 elaborates on the potential open issues and research gaps for WPT in FANETs. Finally, Section 8 concludes the paper and summarizes the future work.

**Figure 2:**
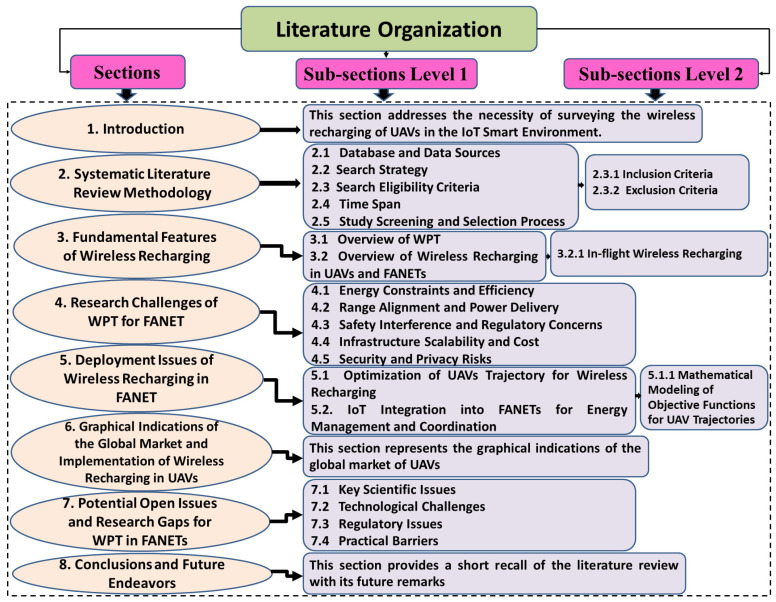
Organizational chart of the review article.

The complete survey organization is shown in Figure 2.

## 2. Systematic Literature Review Methodology

This work conducts a comprehensive literature review to identify peer-reviewed studies on wireless charging solutions for FANETs in IoT-enabled smart environments. The review methodology followed the well-established guidance of the PRISMA recommendations [34].

Probing deeper to ensure the transparency of the systematic literature review (SLP), a structured outline about computing and software engineering was also tracked during the entire research [35].

### 2.1. Databases and Data Sources

The literature review involved a search across a selection of academic electronic databases, including IEEE Xplore, Scopus, Web of Science, Google, and the ACM Digital Library. These databases were selected because of their extensive range of high-impact publications in the fields of computer science, networking, and UAVs.

### 2.2. Search Strategy

Both manual and automated search strategies were used to collect relevant studies. The search string was crafted through an iterative procedure using Boolean operators (OR, AND) and customized to fix the syntax of each database. The primary core domains included: UAV/drone/FANET and wireless charging/energy harvesting/in-flight wireless recharging/charging station/UAV trajectory optimization and Internet of Things/IoT and Flying Ad Hoc Networks, which were applied to keywords, titles, and abstracts.

*KEY-TITLE-ABS ((“UAV” OR “Drone” OR “FANET”) AND (“Wireless Charging” OR “Energy Harvesting” OR “In-flight Wireless Recharging” OR “Charging Station” OR “UAV Trajectory Optimization”) AND (“IoT” OR “Internet of Things”) AND (“Flying* Ad Hoc *Network”))*

The first part of the string (UAV, drone, FANET) depends on the middle part of the string (wireless charging, energy harvesting, in-flight wireless recharging, charging station, UAV trajectory optimization), along with its application in the last part of the string (IoT, Internet of Things, Flying Ad Hoc Networks).

### 2.3. Search Eligibility Criteria

#### 2.3.1. Inclusion Criteria

- The primary search counted in the peer-reviewed journal articles, scholarly books, and full conference proceedings
- Research study that specifically investigates the wireless charging methods for UAVs in FANETs under IoT smart environments
- Empirical results of simulation, trajectory optimization algorithms/methods evaluation, and their mathematical modeling
- Full articles written in English
- Articles published after 2011

#### 2.3.2. Exclusion Criteria

- Non-peer-reviewed sources such as blogs, preprints, white papers, non-scholarly publications, non-scholarly books, meta-analyses, and editorials
- The study does not primarily address the wireless charging of UAVs in FANETs under IoT smart environments
- Duplicate records of the articles
- Papers that are solely theoretical or conceptual without any form of evaluation, as well as previous versions of the same research

### 2.4. Time Span

The literature spanning from 2011 to 2025 examines the progression of wireless UAV recharging within FANETs/IoT, highlighting significant advancements in wireless recharging and IoT integration.

### 2.5. Study Screening and Selection Process

- **Identification:** The initial database search retrieved a total of 275 records, with 20 from ACM, 105 from IEEE Xplore, 50 from Scopus, 45 from Google, and 55 from the Web of Science Digital Library.
- **Duplication Removal:** At this phase, 109 duplicate records were removed to obtain 166 unique entries for the next step.
- **Screening:** The keywords, titles, and abstracts of the remaining 166 records were reviewed to screen the 18 records.
- **Eligibility Assessment Criteria:** During this step, 10 records were removed as they failed to satisfy the eligibility criteria, resulting in 138 articles being selected for full-text evaluation. Among these, 18 were excluded for the following primary reasons: 6 were out of scope, 8 lacked empirical or simulation results, and 4 had insufficient methodological details.
- **Final Selection:** The final selection of relevant studies consisted of 120 papers, which were incorporated into the qualitative review.

**Figure 3:**
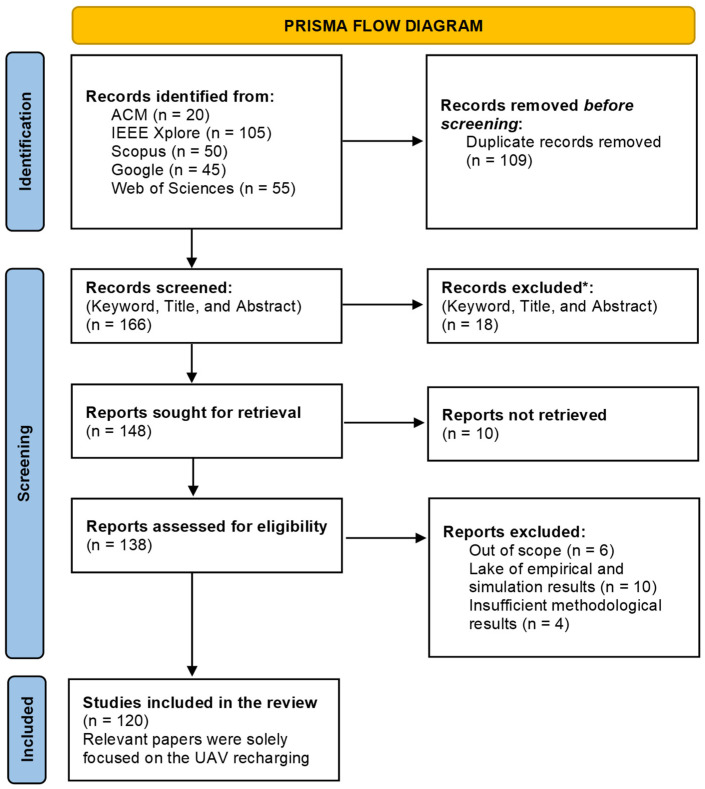
PRISMA workflow chart.

All PRISMA workflow processes and screening methods are demonstrated in Figure 3.

## 3. Fundamental Features of Wireless Charging

### 3.1. Overview of WPT

Wireless power transfer is classified into two main categories based on the distance over which power is transmitted. For power transfer that is efficient over distances shorter than one meter, near-field transmission methods such as Capacitive Power Transfer (CPT), Inductive Power Transfer (IPT), and magnetic resonant coupling (MRC) are applied. Conversely, for power transmission over greater distances, far-field techniques such as laser-based charging and microwave power transfer (MPT) are utilized. A broad overview of the WPT technology literature is provided in Table 1.

The concept of transmitting power wirelessly through air is not novel and has captivated scientists worldwide since the dawn of the 20th century. In 1905, Nikola Tesla obtained a patent for a device designed to transmit intelligible signals or power through a natural medium [53]. This idea has fueled a century-long quest to advance wireless power transfer technology. In 2007, Andre Kurs and his colleagues at the Massachusetts Institute of Technology (MIT) achieved a breakthrough by wirelessly transferring 60 W of power to light up a bulb, heralding the advent of WiTricity [54].

**Figure 4:**
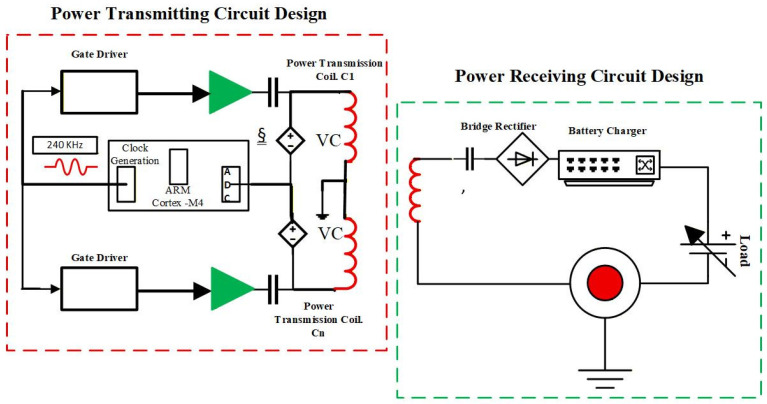
Primary circuit design of power transmission and receiving.

Therefore, the basic power transmission and receiving circuit design can be understood from Figure 4.

A comparison of the WPT method clearly demonstrates the early research on WPT. Table 2 lists the near-field and far-field WPT methods.

### 3.2. Overview of Wireless Recharging in UAVs and FANETs

Energy constraints introduce another layer of complexity that is directly linked to both stability and obstacle avoidance. UAVs are inherently limited by the installed battery capacity, and frequent evasive maneuvers or extended network relaying can accelerate energy depletion, leading to instability or node failure. To address this issue, static charging stations can support periodic refueling during missions, whereas mobile charging platforms and UAV-to-UAV wireless power transfer enable dynamic recharging in the field [23]. A UAV-enabled WPT setup is also analyzed for wireless powered mobile edge computing (MEC) and Wireless Powered Communication Networks (WPCNs). This study examines the deployment of a single UAV to recharge multiple ground stations (GDs) and the coordinated operation of multiple UAVs to service various GDs. The objective was to optimize the efficiency of UAVs’ trajectories [55]. Similarly, a non-convex optimization problem is formulated to handle the constraints of data forwarding, energy renewal, and Quality of Service (QoS). In this methodology, a UAV transfers energy to ground users through Radio Frequency (RF) technology and collects data that are further transmitted to the base station [56]. Ali et al. developed an innovative folding charging point. They applied IPT technology by integrating the receiving coils into the legs of the receiving drones and positioning the transmitting coil at a stationary charging dock [57]. Another study devised a method for in situ power transfer to recharge hotspot UAVs using a flying transmitter UAV (tUAV). Utilizing a Q-learning approach, the flight path of the power source was optimized as a Markov Decision Process (MDP) problem [58]. Eventually, a Multiple-Input–Multiple-Output (MIMO) antenna is suggested for recharging the receiver UAV (rUAV) using RF power transfer. A Proximal Policy Optimization (PPO) was developed to determine the path of the flying source or transmitter UAV (tUAV), enhancing the effectiveness of energy transfer [59]. A Hybrid-action Drone Mobile Charger (HaDMC) was designed to recharge drones at a designated fixed location. Specifically, they employed deep reinforcement learning to develop and train a latent continuous action decoder to enhance drone longevity [60]. Recently, Jian et al. explored the concept of Autonomous Aerial Vehicle Replenishment (AAV-R), which refuels the energy of an Autonomous Aerial Vehicle Task (AAV-T) by connecting with it at the boundary. After transferring energy to an AAV-T, each AAV-R requires a charging station to recharge its battery [61]. These studies state that all drones must return to the ground station for battery renewal. During this period, the area where they were deployed experienced a service interruption or the drones did not deliver quality work [62].

Furthermore, integrating wireless charging strategies with obstacle-aware routing ensures that detours for collision avoidance or link maintenance are energy-efficient, thereby supporting long-term mission reliability. Robust control methods, such as NMPC, provide the foundation for safe evasive maneuvers, advanced routing protocols to maintain connectivity during dynamic operations, and wireless charging strategies to ensure mission sustainability [63].

By combining these approaches, FANETs can achieve resilient, adaptive, and energy-aware performance in complex and unpredictable environments, enabling their deployment in critical applications such as disaster response, urban surveillance, and cooperative monitoring of the environment.

**Figure 5:**
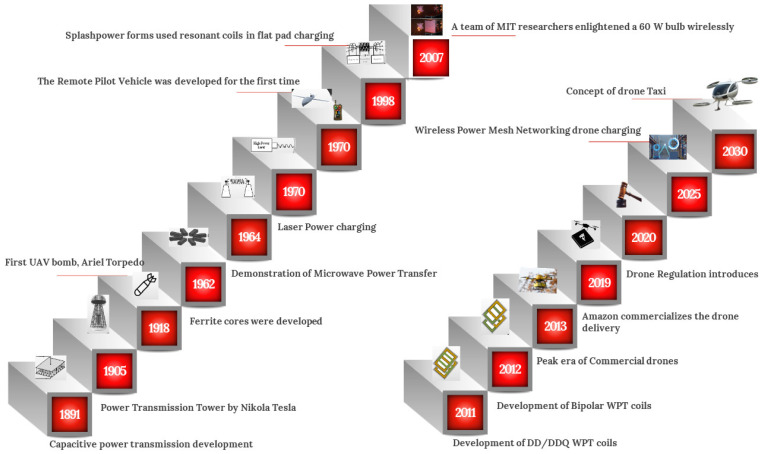
Evolution of WPT charging.

The evolution of WPT charging is presented in Figure 5, and Table 3 provides a demonstration platform for UAV wireless recharging at a glance.

#### In-Flight Wireless Recharging

Mobile wireless charging has recently gained attention as a method for energy replenishment, especially when a component of the charging system is in motion [70,71]. Moreover, recharging an airborne UAV using a discharging UAV (D-UAV) [22] serves as a classic example of mobile wireless charging. Furthermore, the wireless mobile charger is an auspicious approach for achieving on-demand and fully automated UAV battery recharging. This groundbreaking method of in-flight wireless recharging eliminates the need for UAVs to land and connect to stationary charging stations, thereby enhancing their operational effectiveness and extending their flight durations. The concept of in-flight mobile wireless charging for UAVs presents new opportunities for extended missions in remote or inaccessible regions, where conventional charging facilities may be scarce or unavailable, although the range of each in-flight wireless recharging technique is different. Table 4 represents recent breakthrough methods for in-flight wireless recharging of UAVs or FANETs.

## 4. Research Challenges of WPT for FANETs

Despite the significant potential of wireless charging solutions to extend the operational range and autonomy of FANETs in IoT-enabled environments, several challenges hinder their widespread adoption [49]. The effectiveness of these systems is constrained by technical factors, such as limited power transfer efficiency, short charging ranges, and precise alignment requirements of several wireless power transfer methods [82,83]. Operational considerations, including the added weight of charging hardware on UAVs, electromagnetic interference, and compliance with safety and regulatory standards, further complicate their deployment [84]. Moreover, establishing a scalable and cost-effective charging infrastructure in dynamic, large-scale smart environments presents logistical and economic barriers. These constraints underscore the need for ongoing research into more efficient, safe, and adaptable charging methods specifically designed for the unique mobility and energy requirements of FANETs.

### 4.1. Energy Constraints and Efficiency

UAVs operating within FANETs are small and lightweight, which is a design necessity that limits their payload capacity and, consequently, the size and weight of the attached batteries. This inherent constraint results in a minimal battery capacity, which significantly restricts the flight duration, mission range, and ability to support energy-intensive sensors and communication equipment [85,86]. Although wireless charging offers a capable means of extending operational time without requiring frequent battery swaps or physical docking, its practical effectiveness is hindered by the low efficiency and limited energy throughput of current wireless power transfer technologies. These bottlenecks become especially critical when attempting to recharge UAVs in midair or during short landing intervals because the delivered power may be insufficient to sustain prolonged missions or to meet the high instantaneous energy demands of certain onboard systems. Consequently, despite its potential, wireless charging remains a supplementary rather than a primary energy solution for FANET applications in their current state [16].

WPT technologies, particularly those based on RF transmission, face inherent efficiency challenges that limit their practical applications in FANETs [87]. RF-based systems tend to suffer from significant energy loss during transmission, with their efficiency decreasing rapidly as the distance between the transmitter and receiver increases [88,89]. This issue is further compounded by the sensitivity of these systems to alignment; even slight deviations in the orientation or position of the UAV relative to the charging source can result in substantial reductions in power transfer efficiency [90]. In dynamic FANET environments, where UAVs are constantly moving and may experience unpredictable positional shifts owing to wind, turbulence, or navigation requirements, maintaining the precise alignment necessary for optimal energy transfer is challenging. These factors not only reduce the net energy delivered to the UAV but also lead to increased transmission power requirements, higher operational costs, and potential interference with other wireless systems operating in the same frequency bands [91].

### 4.2. Range Alignment and Power Delivery

Near-field wireless charging methods, such as inductive and magnetic resonance couplings, are widely recognized for their high efficiency over short distances. However, their efficacy depends heavily on maintaining a tight coupling and precise alignment between the transmitter and receiver coils [57]. Even slight deviations in position, tilt, or orientation can lead to substantial drops in charging efficiency, whereas increases in separation distance cause the power transfer capability to degrade sharply. In static or controlled environments, alignment can be managed easily; however, in FANET applications, where UAVs are constantly in motion and subjected to environmental disturbances such as wind or turbulence, maintaining this level of precision becomes a major operational challenge. This sensitivity not only limits the practicality of near-field methods for dynamic charging scenarios but also restricts the spatial flexibility required for seamless integration into IoT-enabled smart environments [92].

UAVs, particularly those in FANETs that perform complex sensing, communication, and navigation tasks, require more power than static or low-consumption IoT sensors [93]. Delivering sufficient power wirelessly to sustain such operations without sacrificing transfer efficiency is a non-trivial problem [94]. Increasing the transmission power to meet UAV demand can lead to higher energy losses, increased heat generation, and potential electromagnetic safety concerns while straining the charging infrastructure. In addition, ensuring consistent high-power delivery during short charging windows, such as brief mid-mission stops or aerial hovering, remains difficult with existing WPT systems [95].

Without significant advancements in high-capacity, high-efficiency wireless charging designs, these power transfer limitations will continue to constrain the feasibility of fully untethered long-duration FANET operations in the future.

### 4.3. Safety Interference and Regulatory Concerns

RF-based WPT systems operate by emitting electromagnetic fields to transmit energy over long distances. Although this approach enables flexible, contactless charging, it must comply with the strict regulatory exposure limits set by bodies such as the International Commission on Non-Ionizing Radiation Protection (ICNIRP) [96] and the Federal Communications Commission (FCC). These limits are particularly important in urban or densely populated smart areas, where UAVs in FANETs may operate close to humans, animals, and sensitive electronic equipment such as medical devices. High-power RF transmissions, which may be required to meet the energy demands of UAVs, risk exceeding these safety thresholds, potentially posing health concerns owing to prolonged or repeated exposure to EMFs [97]. Additionally, high-intensity RF fields can interfere with medical devices, wireless communication systems, and complex infrastructure, raising safety and legal compliance issues. Therefore, careful system design, power management, and operational zoning are necessary to balance performance and biosafety requirements [91].

In addition to regulatory and biosafety concerns, WPT systems, particularly those using inductive or resonant coupling, are vulnerable to electromagnetic interference (EMI) from surrounding objects and environmental conditions [86]. Metallic structures, dense building materials, and other sources of electromagnetic noise, which are common in smart urban and industrial settings, can distort the magnetic or electric fields used for energy transfer. This can lead to reduced coupling efficiency, unstable power delivery, and charging interruptions. For FANET applications, where UAVs may need to recharge in cluttered environments such as warehouses, factories, or urban canyons, these interference effects can significantly undermine the reliability and predictability of the wireless charging operations. Addressing EMI susceptibility requires careful placement of charging infrastructure, shielding of sensitive components, and adaptive control systems that can compensate for real-time changes in the electromagnetic environment [98].

### 4.4. Infrastructure Scalability and Cost

Establishing charging stations, whether in the form of ground-based pads, inductive platforms, or directional beam transmitters [99], requires a significant upfront investment in hardware and integration with existing smart city infrastructure. The process often involves modifying physical spaces to accommodate power delivery systems, ensuring network connectivity for monitoring and control, and aligning installations with local energy distribution capabilities. In addition, because FANETs operate on dynamic and often unpredictable flight paths, charging stations must be strategically positioned to be both accessible to UAVs and unobtrusive in the environment. Achieving a balance between accessibility, safety, and minimal disruption to existing infrastructure adds to the overall complexity of deployment [83].

Even when individual wireless charging stations are successfully deployed, scaling the system to meet the operational needs of FANETs remains a major obstacle [100,101]. For UAVs to operate freely and continuously within large-scale IoT-enabled smart environments, wireless charging must be widely available and reliable under varying demand conditions. This entails ensuring adequate coverage across wide geographical areas, accommodating the simultaneous charging of multiple UAVs, and coordinating the charging schedules to prevent congestion or downtime. Large-scale deployments must also contend with challenges such as energy distribution across the network, real-time UAV tracking for dynamic charging allocation, and redundancy planning to maintain service in the event of station failures. These logistical and engineering complexities make scalability one of the most difficult barriers to the widespread adoption of wireless charging for FANET operations [102].

### 4.5. Security and Privacy Risks

Wireless charging systems, which are primarily designed for energy transfer, can inadvertently become sources of information leakage through side-channel attacks [103]. Variations in the current draw, charging cycles, or power consumption patterns can reveal details about a UAV’s operational status, mission profile, or even the type of onboard equipment being used. In IoT-rich FANET environments, where UAVs frequently interact with other devices and infrastructure, such data can be intercepted by malicious actors to infer flight schedules, detect mission-critical activities, and predict UAV behavior. These vulnerabilities are particularly concerning in scenarios involving sensitive operations, such as surveillance, emergency response, or industrial inspection, where operational secrecy is paramount. Therefore, all IoT-rich FANET systems require the ability to ensure appropriate security and manage privacy risks [104]. Without proper encryption, obfuscation, or anonymization of charging-related data, wireless power transfer systems risk becoming an overlooked but exploitable entry point for cyber–physical attacks [105].

FANETs typically rely on a complex ecosystem of IoT devices, edge computing nodes, and smart infrastructure for navigation, coordination, and data exchange purposes. Integrating wireless charging capabilities into this ecosystem without introducing new security vulnerabilities is a significant design challenge. Charging systems may require networked control, authentication protocols, and remote monitoring, all of which can create additional attack surfaces if they are not properly secured. For example, a compromised charging infrastructure can be exploited to disrupt UAV operations, manipulate energy delivery, and inject malicious firmware updates [106]. Furthermore, ensuring interoperability between heterogeneous charging technologies and communication protocols adds another layer of complexity to FANETs [107]. To address these risks, the integration of wireless charging must be approached with the same level of cybersecurity diligence as communication systems, employing robust authentication, intrusion detection, and secure data-handling measures throughout the charging network.

## 5. Deployment Issues of Wireless Recharging in FANETs

In addition to the aforementioned studies, two main research challenges must be addressed to overcome the limitations of WPT for FANETs. In this section, we present the optimization of UAVs and IoT integration issues.

### 5.1. Optimization of UAV Trajectories for Wireless Recharging

The task of optimizing UAV trajectories for wireless recharging involves designing flight paths that maximize energy transfer to ground devices while minimizing UAV energy consumption. This complex challenge requires careful consideration of several factors, including the battery capacity, recharging efficiency, flight dynamics, and spatial arrangement of ground nodes that require power. A key difficulty in this optimization process is finding the right balance between the coverage area, recharging duration, and UAV endurance [108].

To address these multi-objective optimization challenges, advanced algorithms such as genetic algorithms, particle swarm optimization, and reinforcement learning techniques are commonly used. These methods aim to discover optimal or near-optimal solutions that adhere to constraints related to UAV capabilities, regulatory standards, and the specific requirements of wireless charging networks. Moreover, the ability to adapt flight paths in real time based on the changing power demands of ground nodes and environmental conditions is a growing area of research that aims to enhance the overall efficiency and reliability of UAV-based wireless charging systems.

#### Mathematical Modeling of Objective Functions for UAV Trajectories

The objective functions for UAV trajectories are typically calculated using mathematical formulations for handling the optimization issue. The state-of-the-art existing literature is described below.

- In the proposed Trajectory Optimization of Laser-Charged (TOLC) algorithm [109], T is calculated as the total working time of the UAV using Equation (1).
- In 2023 [110], an algorithm was proposed to employ an Energy Transfer (ET) UAV for recharging all Energy Receivers (ERs). The goal is to maximize the harvested energy $E_{k}$ via Equation (4).
- Another Distributed Multiple-Input–Multiple-Output (D-MIMO) [111] setup is anticipated for a three-dimensional trajectory optimization in Equation (6).
- Similarly, a Trajectory Optimization of Multi-UAVs Algorithm (TOUMA) [112] was projected for minimizing the time taken by a UAV to recharge the entire wireless network in (12).
- Periodic charging schemes were proposed in [113] to recharge the RWSN with a minimum charging delay T. The least delay in charging can be achieved by calculating Equations (16) and (17) in Table 5.

Table 5 provides a detailed comparison of the mathematical modeling of objective functions for UAVs’ flying paths defined in the above literature.

### 5.2. IoT Integration into FANETs for Energy Management and Coordination

The integration of IoT into FANETs has become central to addressing one of the most pressing challenges in UAV operations: energy management. IoT platforms provide the infrastructure for seamless data exchange among UAVs, ground stations, and edge/cloud systems, allowing UAVs to share critical information, such as battery status, energy consumption rates, and mission requirements, in real time [114]. By leveraging IoT-enabled communication frameworks, energy usage across the swarm can be monitored continuously, enabling adaptive decision-making that extends the mission duration and enhances the overall system resilience [115,116].

Traditional FANET routing schemes prioritize connectivity and coverage, whereas IoT-based frameworks incorporate energy awareness into the routing and task allocation processes. For instance, UAVs with higher residual energy can be dynamically assigned more complex or energy-intensive tasks. Simultaneously, low-energy UAVs can be rerouted toward charging hubs or tasked with less demanding operations. Machine learning models hosted on IoT edge servers can further optimize these processes by predicting UAV energy consumption patterns and scheduling missions, thereby reducing the risk of in-flight power depletion [117,118].

Smart charging stations connected through IoT infrastructure can dynamically allocate charging slots, manage queuing, and prioritize UAVs based on urgency and mission criticality. When combined with WPT systems, IoT platforms can schedule aerial refueling or short-landing recharges with minimal disruption to swarm operations. This coordination ensures that UAVs can maintain persistent coverage in large-scale IoT-enabled smart environments such as urban monitoring and industrial inspection. Recent studies have highlighted how IoT-assisted charging orchestration can improve both energy efficiency and reliability in large-scale deployments [4,25,119].

In addition to energy management, IoT plays a crucial role in swarm-level coordination and resilience. UAVs can share positional and energy data to synchronize their movements, avoid collisions, and optimize their collective coverage [120]. IoT platforms also provide the backbone for integrating FANETs with other smart systems, such as edge computing nodes and cloud controllers, enabling the holistic management of energy and data flows. This transforms FANETs into highly adaptive, self-organizing systems capable of long-duration missions with minimal human intervention [121,122].

This growing trend of IoT integration in FANETs by employing small UAVs for different tasks is shown in Table 6.

IoT integration elevates FANETs from energy-constrained, isolated networks to intelligent, adaptive, and sustainable aerial ecosystems. Through IoT-enabled monitoring, predictive analytics, and coordinated charging, FANETs can achieve higher energy efficiency, extended mission endurance, and improved swarm coordination [130]. The growing convergence of IoT, machine learning, and wireless charging technologies indicates that the future of FANETs is increasingly autonomous, scalable, and resilient to the challenges posed by dynamic IoT-enabled smart environments.

Eventually, a comparative analysis of traditional charging in FANETs with the wireless charging of IoT-enabled FANETs was conducted. This analysis demonstrates that traditional approaches restrict limited battery-operated missions of FANETs, whereas WPT architectures enable persistent IoT operations through adaptive recharging. Performance is specifically defined in the research questions as key metrics documented in the existing literature, including flight endurance/consistency (min/h), power efficiency (%), human intervention, and Packet Delivery Ratio (PDR, %).

Table 7 presents a detailed comparison between WPT-FANETs and traditional battery-only FANETs.

## 6. Graphical Indications of the Global Market and Implementation of Wireless Recharging in UAVs

Recent market graphs represent the actual situation and growth of IoT-integrated FANETs along with wireless recharging systems. The increasing trend of drone use in smart environments creates more business opportunities for drone/UAV manufacturers. For example, data on the global demand for firefighting drones were retrieved from Grand View Horizon [132], which will raise the interest of researchers and industries in this field. The data are publicly available; therefore, we present them in a pictorial form to facilitate understanding of their future implications.

**Figure 6:**
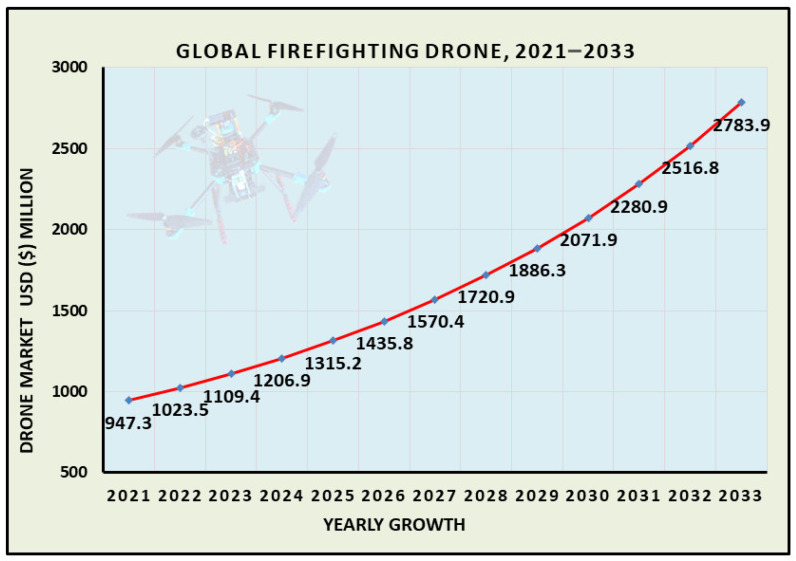
Global firefighting drone market forecasting.

Figure 6 shows the global firefighting drone market.

**Figure 7:**
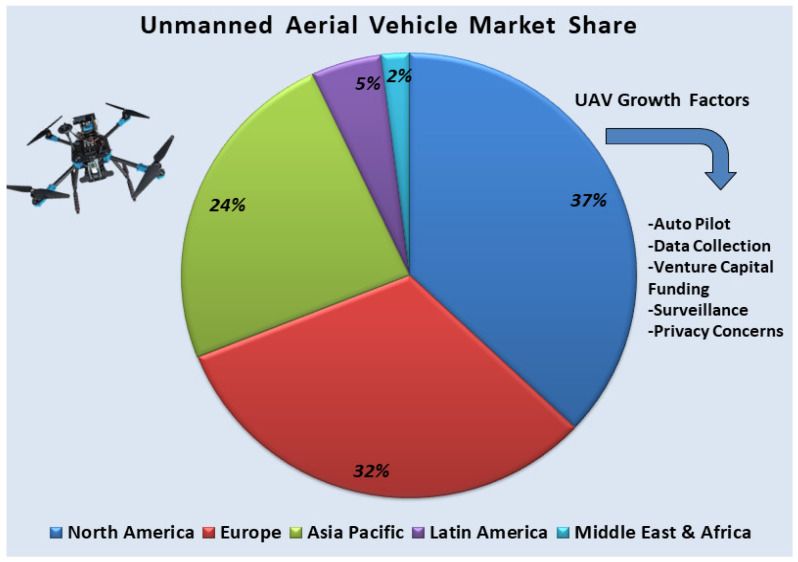
The global UAV market, along with its growing factors.

Similarly, the global UAV market and its growth factors can be accessed through the Precedence Research website [133]. The UAV drone market worldwide was valued at USD 37.68 billion in 2024 and is projected to surpass USD 186.79 billion by 2034, indicating a vast research domain for the future. The overall UAV market size, along with its key growth factors, is also illustrated in Figure 7.

Moreover, the higher implementation of smart homes and cities has become a necessity for surveillance and security services in today’s society. This data will help to understand the gaps in the modeling of UAVs, which are openly available on the Scoop Market [134]. The Global Smart Home Security Market is projected to experience substantial growth, increasing from USD 28.4 billion in 2023 to approximately USD 107.1 billion by 2033.

**Figure 8:**
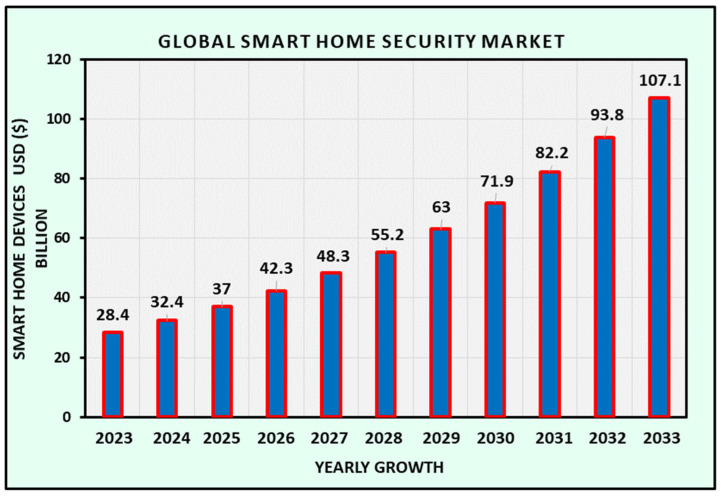
Global smart home security market forecasting.

Figure 8 illustrates the projected IoT market demand.

**Figure 9:**
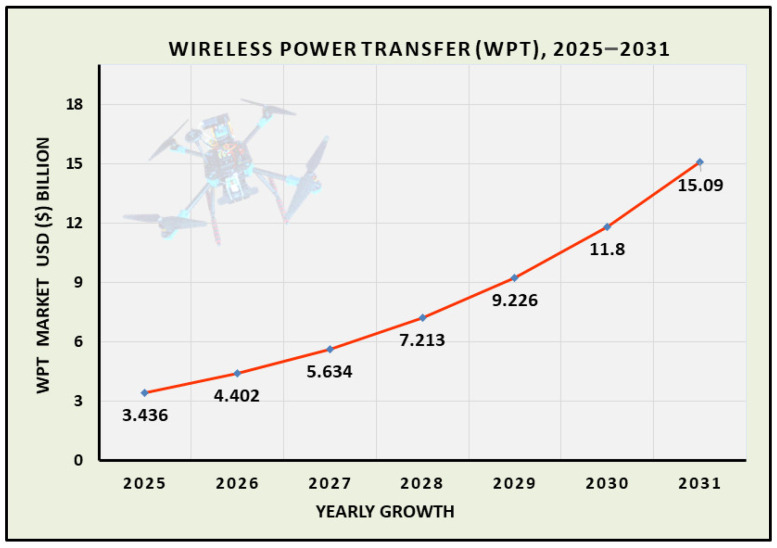
Global market size forecasting of wireless power transfer.

The global market size of WPT is also experiencing remarkable growth and is expected to reach USD 15.09 billion by 2031. This data from Market Report Analytics represents the diverse applications of WPT in the electronics industry in the future [135]. Figure 9 clearly shows the prediction about WPT.

Despite the drone market and IoT demand forecasting, there is still a research gap in the advancement of wireless recharging of UAVs. Current commercial strategies for wireless recharging offer partial support for recharging interfaces and energy management in FANETs. In summary, it is essential to align technical research with the above-presented market forecasting, which will fill the significant research gaps in hardware design, regulatory issues, modeling, and real-world implementations.

## 7. Potential Open Issues and Research Gaps for WPT in FANETs

Expanding on the research challenges and deployment issues outlined in the aforementioned Section 4 and Section 5, this section summarizes them as follows.

### 7.1. Key Scientific Issues

Most existing studies consider simple mobility, operational, and propagation models without considering the highly dynamic nature of UAVs in the 3D airspace of a realistic IoT environment [16,49]. There is an evident demand for intelligent simulation frameworks, data-driven modeling, and energy-aware networks. In addition, the performance of UAVs can be enhanced by integrating online learning algorithms, optimizing UAV trajectories in 3D areas, scheduling recharging, and using tracking techniques for task allocation.

### 7.2. Technological Challenges

From the perspective of technical challenges, the size of UAVs, limited capacity of their payload, and budget for quick recharging in UAVs still need to be improved in the present UAV platforms [85,86]. Open issues for future research should focus on the lightweight manufacturing of UAVs, power allocation strategies for UAV-to-UAV power transfer, coverage-efficient antennas, durable charging protocols capable of maintaining the cooperative performance of numerous flying UAVs, harsh weather conditions, and channel disruptions.

### 7.3. Regulatory Issues

Contactless recharging methods are promising but depend on some vital factors such as aviation regulations, alignment of power transfer to the receiver, human exposure safety, and spectrum rules [84,96,97]. All previous studies on WPT have been explored independently without tailoring the dense IoT environment. Future research clearly demands regulatory agendas that need to be addressed explicitly, such as the use of shared space by UAVs and ensuring the safety measures of high-power voltage transfer in urban and populated infrastructure. Furthermore, cyberattacks, security, and privacy [104,105] concerns can be addressed using jamming, mobile edge computing, and blockchain-enabled UAVs, as well as cooperative communication among UAVs for power splitting.

### 7.4. Practical Barriers

Finally, in terms of practical barriers, the state-of-the-art literature for wireless recharging in UAVs is employed in ideal, small-scale scenarios with diverse application assumptions. Large-scale deployment, energy reservoirs, optimal implications of charging stations, maintenance costs of charging grids, and functionality of logistic fleets of UAVs in far-flung or remote areas are also crucial practical barriers [100,101]. These barriers continue to create a research gap between real-world situations and simulation-based environments. To bridge this gap, future studies should involve large-scale experimental trials, efficient placement of charging stations, and collaboration with the industry for efficient recharging coil designs.

## 8. Conclusions and Future Endeavors

Although wireless charging holds considerable potential for enhancing the endurance and autonomy of FANETs in IoT-enabled smart environments, its implementation faces a combination of technical, operational, regulatory, and security barriers. Current WPT technologies struggle with limited range, low efficiency, and alignment sensitivity. Meanwhile, high energy demands and mobility constraints make it difficult to optimize a reliable mid-mission charging trajectory. Safety regulations, electromagnetic interference, and infrastructure complexity further hinder large-scale deployment. Additionally, the integration of charging systems with communication networks introduces potential cybersecurity vulnerabilities. Overcoming these challenges will require advances in high-efficiency, scalable, and secure wireless power delivery solutions, along with careful consideration of environmental, operational, and regulatory factors to ensure their safe and practical adoption. In conclusion, wireless charging solutions for FANETs represent a crucial stepping stone towards achieving truly autonomous and self-sustaining aerial networks in IoT-enabled smart environments. As this technology matures, it will undoubtedly play a pivotal role in shaping the future of smart cities and interconnected systems, bringing us closer to a more efficient, sustainable, and technologically advanced society.

Additionally, current research on wireless power transfer methods should support the expansion of the geographical area of wireless charging structures and be more adaptable for energy transfer to UAVs and IoT devices.

## Figures and Tables

**Table 1:** WPT implication.

WPT Method	Fundamental Principal	Efficiency	Distance Range	Frequency	Transmission of Power	References
Capacitive Wireless Power Transfer (CPT)	Transfers power using alternating electric fields between conductive plates (capacitors)	High	Millimeters to centimeters	4.2 × 10^−3^ GHz	0.0037 kW	[35,36,37,38]
Inductive Wireless Power Transfer (IPT)	Energy transfers using alternating magnetic fields between the two coaxial coils	High	Centimeters to tens of centimeters	2.2 × 10^−5^ GHz	100 kW	[39,40,41,42]
Magnetic Resonant Coupling (MRC)	Power transfers by connecting the transmitter and receiver coils that are set to the same frequency	Medium to High	Centimeters to meters	6 × 10^−5^ GHz	818 kW	[43,44,45,46]
Laser Beam Power Transfer (LPT)	A laser beam from the ground is aimed at a photovoltaic (PV) cell on the drone. This cell changes the light into electrical power	Medium	Meters to kilometers	2.4 GHz	1 × 10^−3^ kW	[20,47,48,49]
Microwave Radio Frequency (RF) Power Transmission	Microwave radiation is used for power transfer from a phased-array transmitter to a rectifying antenna, or “rectenna,” located on the drone.	Low to Medium	Meters to kilometers	5.8 GHz 2.45 GHz	5 × 10^−2^ kW 4.39 kW	[50,51]
Parity-Time-Symmetric WPT	Power transmission is done by maintaining the Parity Time (PT) Symmetric phase stability	High	Centimeters to meters	1.2 × 10^−4^ GHz	6.5 × 10^−2^ kW	[52]

**Table 2:** WPT technology methods.

WPT Method	Research Proposed	Coil Gap/Beam Type	Coupler Area/Acceleration Time	Passing Medium	Energy Density/Divergence	Power Loss/Line of Sight
CPT	Research was proposed in 1981	<1 mm	Less	Metals	${\pi Ɛ}_{0}{Ɛ}_{r}\left(t\right)E_{c-max}^{2}$	High
IPT	Research was proposed in 1830	>10 cm	High	Only air	$\pi \frac{B_{L-max}^{2}}{\mu_{0}\mu_{r}}$	Low
MRC	Research was proposed in 2007	2 m	High	Body tissue, object metals	-	Low
LPT	Research was proposed in 1970	Concentrated	Long	2.4 GHz	Low divergence	Line of sight required
Microwave RF	Research was proposed in 1964	Non-Concentrated	Short	0.12 MHz	Very high divergence	No line of sight needed

**Table 3:** Wireless recharging demonstration platform for UAVs.

Demonstration System	WPT Method	Device Type	Novel Innovation	Reference
Magnetic Integrated WPT System	Magnetic Resonance Coupling	UAV	A stable and misalignment-tolerant charging system for UAV power transfer through a tiny receiver.	[64]
Automated Wireless Charging Station	Inductive Power Transfer	UAV	A precise landing of a UAV for wireless recharging but with a longer sensing time and lower efficiency.	[65]
Autonomous Charging System	Inductive Power Transfer	UAV	It facilitates adjustable charging for long-range detection, unaffected by varying lighting conditions.	[66]
Autonomous Landing and Charging	Inductive Power Transfer	UAV	It proposed efficient computation time but a higher landing time with a small range.	[67]
Microwave-Powered UAV	Laser Power Transfer	UAV	A micro-UAV achieved perpetual flight by utilizing energy from a laser on the ground, which was aimed at its photovoltaic cells.	[51]
UAV Docking System	Capacitive Power Transfer	UAV	A lightweight, conformal capacitive coupling system that highlights its potential for aerodynamic integration and facilitates charging when briefly in contact with a perch.	[38]
Misalignment Tolerance System	Inductive Power Transfer	UAV	A circular-pad transmitter featuring multiple overlapping coils and a compact receiver was developed to maintain efficiency even when misalignment occurs during landing.	[42]
500 W Wireless Charging System	Orthogonal Magnetic Concept	UAV	A proposed network of charging is based on a polarized transmitter and a U-type flat core that is perpendicular to the receiving coil. This system can transfer 500 W of direct current to the batteries of the UAV.	[68]
A Robotic Mobile Charging Tender	Conductive Charging	UAVs	An electromagnetic system is proposed that supports the UAVs to lock with a ground charging station and creates a physical connection for efficient conductive charging.	[69]
Long-Range Laser Powering	Laser Power Transfer	UAV	The experiment showcased the ability to supply power to a UAV from distances greater than 100 m by a high-power laser in conjunction with an optimized photovoltaic cell array, emphasizing accurate beam tracking and strict safety protocols.	[47]

**Table 4:** Wireless charging pad and in-flight charging of UAVs.

Technology System	Type of Charging	Output Power/Battery	Dimensions of Charging Pad/Alignment	Technology Implication	Key Features	Charging Distance	References
WiBotic	Wireless Charging Pad	100–300 W	91.4 cm × 91.4 cm	Commercial	Commercially available product that is accessible for public use.	<10 cm	[72]
Heisha	Wireless Charging Station	17.5 V, 6 A maximum	80 cm × 80 cm	Commercial	Concentrates on worldwide drone charging solutions with industry-leading charging stations.	<10 cm	[73]
H3 Dynamics	Autonomous Charging Station	12 V, 17.5 A maximum	2 cm × 2 cm	Commercial	An advanced autonomous charging system used for critical mission operations.	<10 cm	[74]
Power Republic Corporation	Wireless Power Transfer	200 W	-	Commercial Android/iOS consumer-focused)	Emphasis on WPT charging solutions for drones.	<10 cm	[75]
GET Corporation	In-flight Charging	12 × 10^4^ W	Circular coils less than 6 m in diameter	Commercial	While in flight, the drone charges for 6 min, increasing its flight duration to 25 min.	3 m	[76]
GuRu	In-flight Charging	>500 W	90 cm × 90 cm	Commercial	Operating at 24 GHz, it works on high-frequency millimeter-wave radio signals, the same as the frequency used in 5G networks.	30 feet, near 9 m	[77]
Laser Power Beaming	In-flight Charging	2 × 10^3^ W	Strict alignment	Laboratory experiment	It provides long-range laser beaming power transfer to micro-UAVs	100 m–1 km	[78]
Drone Charging System	Wireless Charging Pad	100 W	Precise alignment	Laboratory demonstrations	Usually used for short-range UAV battery charging.	25 mm	[79]
Air Core Beam ATR Japan	In-flight Charging	3–5 V	Depends on position	Demonstration at Wireless Technology Park, Japan	Power transmission is done by an air core beam (radio waves) to enlighten the drone’s LEDs.	Medium range	[80]
Reach Wireless Power DARPA	In-flight Charging	50 W	Controlled coordinates	Demonstration for DARPA	Multiple power transmitters work in unison, creating a mesh network for recharging the drone.	6 m	[81]

**Table 5:** Mathematical modeling of trajectory optimization for wireless recharging by UAVs.

Research Year	Objective of Optimization	Proposed Algorithm	Mathematical Modeling	Reference
2022	Optimize the flight trajectory of the UAV. Plan the travel of Mobile Unmanned Vehicle (MUV). Minimize the UAV’s total working time (T). Ensures that every sensor maintains the threshold energy level. Guarantees the UAV’s energy to recharge sensors.	Trajectory Optimization of Laser-Charged (TOLC) UAVs for charging WRSNs. The TOLC is an NP-hard problem.	(1)T=L(U)vf+ ∑tiq∈TQtiq+ ∑tjg∈TGtjg+ ∑tjw∈TWtjw In the above, $L\left(U\right):$ the length of the flight path; $v_{f}\;$: the flight speed of the UAV; $t_{i}^{q}\;$: the hovering time to recharge sensors at $q_{i}\in \;Q$; $t_{j}^{g}$: the hovering time to be recharged by MUV at $g_{i}\in \;Q$; ${\;t}_{j}^{w}$: the MUV waiting time at a point $w_{i}\in \;Q$. RF wireless power transfer model is as follows: (2)Pqi,sjT=ζ⋅hj,i⋅PR,hj,i=d2qi,sjγ0 In the above, d is the distance between the sensors and UAV hover.	[109]
2023	Deployed an ET UAV to transmit power to all ERs. Maximize the total energy harvested by all ERs during a specific mission period. Optimize the Trajectory of UAV ET. To achieve a balanced tradeoff between the UAV’s energy consumption and the overall WPT performance.	Total harvested energy was calculated based on the calculus idea. The harvested energy $E_{k}$ is a non-concave function of the UAV’s trajectory, which makes the optimization problem non-convex and difficult to solve.	(3)max{xt, yt, h(t)}∑k=12Ek In the above, $E_{k}$ is the total energy harvested by ER *k* during the entire mission period T. This energy is calculated by integrating the power received by the ER over time in (4)–(5): Ekxt, yt, ht(4)= ∫0TQk xt, yt, htdt In the above, $x\left(t\right),\;y\left(t\right),\;h(t)$ is the power harvested by ER *k* at a time t. This time t is defined as follows: Qkxt, yt, ht= ηPβ0h2t+ (xt− xk)2+ (yt− yk)2(5)=ηhk(t)P	[110]
2023	A 3D trajectory system is introduced in a D-MIMO infrastructure. Optimizing the 3D trajectories of the Mobile Access Points (MbAPs) and their transmission power. Optimization problem is calculated to enhance the Quality of Service (QoS) for the user with the weakest link. To ensure fairness among users by maximizing the minimum ergodic rate lower bound for all users.	The optimization problem is formulated as a max–min problem. The proposed online trajectory system is based on Space Division Multiple Access (SDMA). The main problem is divided into two sub-problems via Block Coordinate Descent (BCD). The problem is non-convex in nature.	(6)maxQ, P min kR˜kn This main problem is subject to several constraints: Maximum Transmit Power: Equation (7) enforces a maximum power constraint for each MbAP’s transmission. (7)∑k=1Kpm, k[n]≤ Pmax, ∀m Maximum Travel Distance: Equation (8) limits the distance an MbAP can travel within a single timeslot. (8)qmAP n− qmAP n−12 ≤Dmax ∀m Collision Avoidance: Equation (9) maintains a minimum safe difference $\left(D_{min}\right)$ between any two MbAPs to prevent collisions. (9)qmAP n− qḿAP n2 ≥Dmin ∀m≠ ḿ UAV—User Safety: Equation (10) ensures a minimum safe distance $\left(D_{safe}\right)$ between MbAPs and users. (10)qmAP n− qkU n2≥Dsafe ∀m, k Altitude Constraint: Equation (11) restricts the MbAPs’ altitude to avoid issues related to Earth’s curvature, which is a specific constraint for 3D optimization. (11)e3TqmAP n ≤Hmax, ∀m,	[111]
2025	It is trajectory optimization of multi-UAVs and multi-MUVs for charging Wireless Rechargeable Sensor Networks (WRSNs), known as the TOUM problem. To design an optimal cooperative travel plan for multiple UAVs and MUVs to charge the WRSN. To minimize the time taken by the UAV that requires the most time among all UAVs. Ensuring the energy level of every sensor in the network is at or above a specified threshold.	Discover the multiple UAV-based Traveling Sales Person (TSP) problem first. They proposed a TOUM Algorithm (TOUMA). Investigated a TOUM Deep Q-Network (TOUM-DQN) algorithm to extract information that forms the intelligent UAV and MUV travel path. The problem TOUM is NP-hard in nature.	(12)minT=max{TkUk∈U} In the above, $T_{k}$ is the total time consumption for UAV, $u_{k}$ calculated as in (13): Tk=d(Fk)vf+∑pik∈Pktip, k(13)+ ∑bik∈Bktib, k +∑bik∈Bktiw, k The complete mathematical formulation is given by (14): (14)min∑k=1mTk The total objective function for the MUTSP is defined as (15): (15)minf= α.dmax+ β.∑k=1mFk subject to various constraints for UAV paths and visits. In the above, $d_{max}$ is the distance covered by the UAV with the longest trajectory. ${d(F}_{k})$ is the distance of the trajectory for UAV $u_{k}$. α and β are weighting coefficients.	[112]
2024	The objective of the problem of periodic charging with minimum charging delay in a WRSN. Two periodic schemes for recharging are proposed. These are the PAD-based Charging Scheme (PBC) and Sensor Node Charging Scheme (SNBC). To identify a charging loop that minimizes the total charging delay. Ensuring all sensor nodes are charged.	The optimization goal was to minimize the charging delay, denoted as T. T represents the total time for a charging cycle. This includes the time spent flying along various sub-paths and the time spent recharging the UAV at charging stations. The problem is NP-hard in nature.	(16)minT (17)T= ∑i=0M∑j=0M∑x=1SEQi, jdi,j,x ti,j, x+const $d_{i,j,x}$ is the is a decision variable that is 1 if the UAV flies along the sub-path $SEQ_{i,j,x}$ and 0 otherwise. $t_{i,j,x}$ is the time cost for the UAV to fly along the sub-path $SEQ_{i,j,x}$. The main problem is subject to several constraints: **UAV Energy Limit:** $C_{i,jx}\leq E_{UAV},\forall_{i,jx}\;\;\;\;(18)$ **Node Charging Needs:** Charging needs of nodes can be seen via Equations (19) and (20). $l{⋃}_{i,j,xs.td_{i,jx}=1}S_{i,jx}=S$ (19)∑i,j,xs.tsk∈Si,j,xdi,jx=1,∀Sk∈S **Charging Cycle Path:** The UAV’s journey must start and end at the base station (BS), denoted as $p_{0}$. **Node Survival:** Each sensor node’s battery must not be depleted before it is recharged, as in (20). (20)rkt>0, ∀k, t	[113]

**Table 6:** IoT Integration into FANETs by employing UAVs.

Research Year	Application/Implementation Focus	Key Contribution	Shortcomings	Reference
2024	A secure and efficient authentication framework for Internet of Drones (IoD)	PAF-IoD system checks user identity using three methods. These are the AEGIS encryption method, XOR operations, and the SHA-256 hash function to make drone–user interactions more secure and private.	Absence of protection against physical capture, node capture attack, and forward secrecy.	[123]
2020	Routing protocol for FANETs	It is an energy-efficient routing protocol modified by the AntHocNet protocol. An innovative ‘Energy Stabilization Threshold’ (es_threshold) was introduced to save energy and enhance the lifetime of networks.	The performance of AntHocNet is only validated under Random Way Point rather than realistic patterns.	[124]
2025	Energy-monitoring system of buildings	It proposes an optimized Artificial Neural Network (ANN)-based predictive framework specifically for energy management.	The data set was collected from Kaggle, which is limited to a few geographical areas.	[125]
2023	Detection of Sybil attacks in IoFT	The scheme finds Sybil attacks by using data from UAV radio signals. It uses physical layer data like RSSD and TDoA. It can spot both regular and smart malicious nodes in the Internet of Flying Things (IoFT).	Before using the system, more tests may be needed, like how interference and blockages affect it, as well as networks with many nodes close together.	[126]
2023	Energy-efficient data routing in FANET-assisted rechargeable IoT Network	An optimization problem formulated to reduce both the energy used by UAVs and the information loss in IoT networks. A Q-learning-based data forwarding scheme (QDFS) is suggested to find the best path for data to travel from the source to the base station.	This study did not limit the speed of relay UAVs. As a result, they might move out of the line of sight (LoS) range, leading to information loss.	[127]
2021	IoFT can work for Flying Cloud, Edge, Fog Computing, and Flying cellular networks.	This survey focuses on the main features of the Internet of Flying Things (IoFT). It compares IoFT with Flying Things (FT) and IoT. The study classifies important IoFT applications. It introduces a new way to organize existing IoFT-related studies.	Energy consumption always remains a significant limitation for both UAVs and IoT.	[128]
2022	Environment monitoring via FANETs	A new algorithm called Unmanned Aerial-AntHocNet is proposed. It was compared with existing routing protocols. The Random Waypoint mobility model was used in tests to mimic a specific flight pattern for each UAV of a FANET.	FANETs face challenges due to their high mobility, low UAV density, and frequent changes to their topologies, which make communication difficult.	[129]

**Table 7:** Comparison between traditional FANETs and WPT IoT-enabled FANETs.

Performance Metric	Traditional FANETs Charging	WPT IoT-FANETs	WPT Charging Advantage	Reference
Flight endurance/consistency	120–60 min	2–6 h	Very high flight endurance	[19,52,82]
Power efficiency%	75–90%	65–85%	High charging feasibility	[43,44,82]
Human Intervention	Yes	No	No need to land, recharging on demand	[45,46,82]
PDR%	75–88%	82–95%	Less PDR due to continuous power supply	[127,131]

## Data Availability

Data are contained within the article.
